# The Interactions Between Antibiotic Resistance Genes and Heavy Metal Pollution Under Co-Selective Pressure Influenced the Bio-Enzyme Activity

**DOI:** 10.3389/fchem.2021.691565

**Published:** 2021-07-14

**Authors:** Zheng Qi, Yue Qi, Zhiwei Le, Furui Han, Fang Li, Hong Yang, Tielin Zhang, Yajie Feng, Rijia Liu, Yuan Sun

**Affiliations:** ^1^ Engineering Research Center for Medicine, Harbin University of Commerce, Harbin, China; ^2^ Technology Center of Harbin Customs, Harbin, China

**Keywords:** heavy metals, antibiotic resistance genes, enzyme, geographic information system, dairy farm

## Abstract

The spread of antibiotic resistance genes (ARGs) has brought potential risks to public health. However, the interactions between heavy metals and ARGs, as well as their potential effect on bio-enzyme activity under the pressure of co-selectivity in soil still remain poorly understood. In this work, the distribution characteristics and the co-selective relationship of 28 ARGs and eight heavy metals in soil in a dairy farm were visualized *via* the geographic information system (GIS) technique. Eight kinds of heavy metals were detected by an atomic fluorescence spectrometer and atomic absorption spectrophotometer, which were further evaluated *via* the single factor pollution index value. The GIS analysis showed that arsenic (As) was the key element responsible for soil pollution, which was found to be positively related to soil depths. The top three comprehensive scores of ARGs ranked the orders of *sul2* > *tetX* > *blaTEM*, indicating the high potential of risk caused by these genes in the soil environment. In addition, the functional predications performed with the 16 SrDNA sequencing data based on the KEGG database indicated that the sulfonamides in soil involved multiple pathways, especially the metabolism, transport and catabolism, and membrane transport processes. This suggested that most bio-enzymes were found to be expressed in low activities in different pathways. Significant correlations were observed between the heavy metals and ARGs (*p* < 0.05), particularly between the ARGs and As, Cu, Ni, Pb, and Zn (*p* < 0.01). This study offers deep insights into the potential interactions between heavy metals and ARGs in soil and provides guidance for the fabrication of enzyme-based smart materials for soil remediation in dairy farms.

## Introduction

Enzyme-based smart materials have received worldwide attention in the past few decades, however, knowledge concerning the dynamic characteristics of bio-enzymes encoded by antibiotic resistance genes (ARGs) influenced by the heavy metals in soil still remains poorly understood, although these biological enzymes would have promising use in the fabrication of smart materials. Considering this, this study aims to investigate the changes in the activities of bio-enzymes as influenced by the co-selective pressure of ARGs and heavy metal pollution in soil in the context of the contents of heavy metal and relative abundance of ARGs. In fact, a large number of antibiotics have been widely used for the purpose of disease control in human and animals, while most of the antibiotics still remain un-metabolized after digesting by animals. Antibiotics in the gut can selectively sort the antibiotic-resistant bacteria with antibiotic resistance genes (ARGs), making livestock feces an important repository for antibiotic residues and ARGs (Qian et al., 2016; Zhang et al., 2018). Antibiotics accumulated in the environment can lead to selective pressure on microbial antibiotic resistance (Zhan and Xiao, 2017). All kinds of microorganisms are important hosts of ARGs. One of the important sources of ARGs in the environment is the potential internal resistance of environmental microorganisms and exogenous input. ARGs can spread widely in the environment through a variety of media, directly or indirectly transferred through pathways, inducing the appearance of varieties of resistant bacteria in the process of transmission.

Previous studies highlighted that the overuse or misuse of antibiotics in livestock, aquaculture, and agricultural production had been the major source to the increased amount of ARGs (Zhou et al., 2016; Duan et al., 2019). Environmental antibiotic residues often have high selective pressure on bacteria, which can significantly increase the probability of ARGs acquisition by bacteria and thus accelerate the diffusion of ARGs through the horizontal transfer route (Gilling and Stokes, 2012). For example, the co-selection of ARGs provoked by heavy metals has become an emerging environmental problem (Berendonk et al., 2015; Pal et al., 2017). In comparison to antibiotics, heavy metals are non-biodegradable species that can put long-term selective pressure on bacteria to generate ARGs (Song et al., 2017). Moreover, long-term heavy metal exposure can also lead to an increase in diversity and abundance of the ARGs in soils (Hu et al., 2016a; Hu et al., 2016b).

There are various kinds of soil enzymes in the ecosystem, such as catalase, which are enzymes that can promote hydrogen peroxide to decompose to harmless wastes, and high content of catalase that can greatly affect the ability of hydrogen peroxide in the decomposition and metabolism process of soil. The hydrolases, such as sucrose enzymes, are important enzymes in the physiological metabolism of sugar, and the content of the enzyme can affect the metabolism of soil, which can indirectly reflect heavy metal contamination in soil (Spohn and Kuzyakov, 2014). The activities of the oxidoreductase and hydrolytic enzyme decreased until the function of the soil cell disappeared. Kandeler et al. (1999) found that the activities of the oxidoreductase and hydrolytic enzyme decreased in soil polluted by excessive zinc, copper, nickel, and cadmium, among which the activity of urease, alkaline phosphatase, and xylanase decreased significantly. The original function of enzyme activity in soil can be changed by heavy metal pollution, resulting in the reduction in microbial species and damage to soil biodiversity (Zhang et al., 2007). The dehydrogenase which can catalyze the dehydrogenation of microorganisms in soil is indicative of heavy metal pollution indexes such as Cd, Cr, Cu, Pb, and Zn. The lower the dehydrogenase content, the more severe the soil pollution (Razavi et al., 2016; Allison and Jastrow, 2006). Catalase is a kind of hydrolytic enzyme, which can be used as a biological indicator for heavy metal pollution of As, Cd, Cr, Cu, Hg, Pb, and Zn. Amylase, alkaline phosphatase, acid phosphatase, arylsulfatase, cellulase, β-glucosidase, acetylglucosidase, and sucrose enzyme, protease, and urease can indirectly reflect the biological indicators polluted by heavy metals in soil (Wang et al., 2007; Roman and Kuperman, 1997).

Considering this, in this work, the distribution features and the potential interactions between the heavy metals and ARGs in soil were investigated. Their accurate spatial distribution of soil was visualized *via* the GIS technique. The contents of the heavy metals were determined *via* the atomic fluorescence spectrometer and atomic absorption spectrophotometer method, and the expression patterns of the ARGs were explored *via* Q-PCR, and their effect on the bio-enzyme activity was also explored. The contamination level of heavy metals was evaluated by the single factor pollution index and the comprehensive pollution level was calculated and measured by Nemerow pollution index. Principal component analysis (PCA) was carried out in order to identify the potential sources of the heavy metals and revealed the underlying impact exerted on the formation of ARGs. The relationships between the relative abundance of ARGs and heavy metal in the soil of a dairy farm were discussed. This study provides guidance for the development of enzyme-based smart materials.

## Materials and Methods

### Samples Collection and Characterization

Dulbert Mongolian Autonomous County (hereinafter referred to as Dumont) is located in the Songnen Plain in the west of Heilongjiang Province. The main landforms include grassland, sand hills, a lake bubble, and saline-alkali land. In terms of the stratigraphic structure, it is dominated by a large block of motley conglomerated with a sedimentary thickness of 62–94 m. The quaternary surface measuring 1.0 m is gray-black humus, and the underlying layer is made up of gray-yellow silty clay which has a flowed sand, motley sand, and gravel layer with a sedimentary thickness of 118 m (Shi, 2004; Du, 2018). It is classified by the European Union and Domestic Green Food Certification Organization as a “green pure land, natural gemstone” natural resource, which has never been polluted. The dairy farm covers an area of approximately 70,000 m^2^ and the milk yield comes from a total of 200 cows, which provides a continuous milk source to a famous milk brand.

The soil samples were collected according to the requirements of the environmental quality standard (GB 15618-2018) and the technical specification for soil environmental monitoring (HJ/T 166-2004). The monitoring scheme (i.e., distribution mode, monitoring items, and sample quantity) was formulated. The sampling points were designed by the GIS grid method, longitude and latitude of every sampling points were confirmed, and then input into the GPS instrument. The on-site sampling points were performed according to the GPS alarm. The samples of the 10 cm-deep soil and the 50 cm-deep soil were accurately taken by a soil drill. Some soil samples were sealed in lightproof bags and put on ice, while the soil samples which were not sealed were used for ARG determination. The labels were marked with sampling time, sampling places, sampling numbers, monitoring items, sampling depths, and in-site longitude and latitude.

**FIGURE 1:**
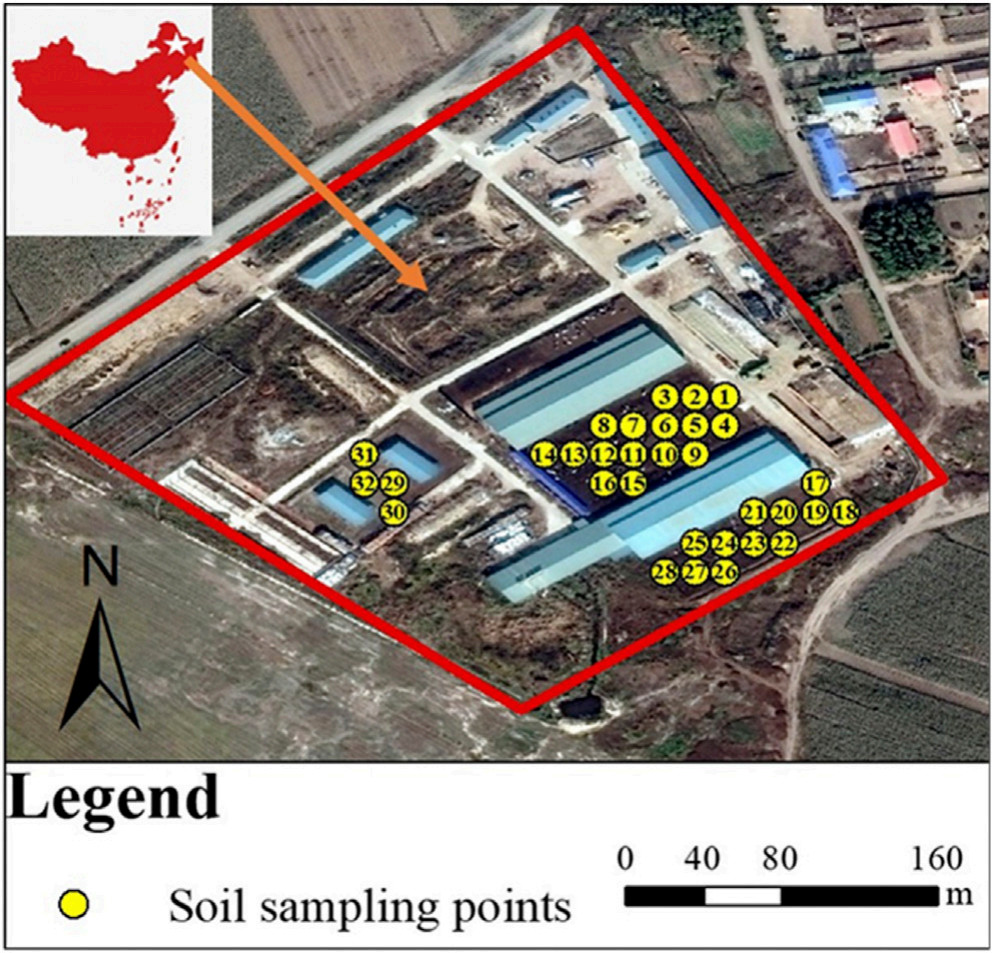
The GIS image showing the exact location and sampling points of the dairy farm in Dumont.

A total of 64 soil samples were collected at the depth of 10 and 50 cm across the dairy farm land of Dumont in June 2019 (Figure 1, Supplementary Table 1). The 10 cm-deep soil was collected from the soil surface, where the bacterial biomass and activity were relatively higher. The 50 cm-deep soil was taken from the subsoil, where the soil layer in the dairy farm was nearly 118 cm in thickness. Three discrete subsamples were collected within 1 m^2^ and mixed to constitute one composite sample of 500 g. The samples were transported to the laboratory within 5 h after sample collection, which were immediately passed through a 0.25 mm (20 mesh) sieve. A total of 0.25 g of each sample was sifted over a 0.25 mm (60 mesh) sieve used for DNA extraction and stored at −80°C prior to ARG measurements. And the rest of the soil samples were screened over a 0.15 mm (100 mesh) sieve and dried naturally prior to pH and heavy metal measurements.

Soil pH was measured by a multi-parameter tester using a pH electrode with a soil-to-water (CO_2_-free deionized) ratio of 1:2.5. Sieved samples were digested, and the concentrations of targeted metals, including Hg, As, Cu, Cr, Cd, Zn, Ni, and Pb, were quantified *via* an atomic absorption spectrometer (AAS, ZEEnit 700P; contrAA 700, JENA) and atomic fluorescence photometer (AFS-9230, JI TIAN). A total of 0.1 g of Cu, Zn, Pb, Ni, Cr, and Cd in the soil were put into PTFE tubes. Then, 5.0 ml of HNO_3_, 2.0 ml of HF, and 1.0 ml of HClO_4_ were added, the tubes were placed in the graphite digestion instrument, covered with the lid, the temperature was adjusted to 120°C for 30 min, then the temperature was raised to 180°C for 3 h. The lid was removed and the solution was left in the tube to be pressed into a nearly dry state, cooled to room temperature, and a constant volume, then Cu, Cr, Cd, Zn, Ni, and Pb were determined by atomic absorption spectrometry. A total of 0.3 g Hg and As were added in a 50 ml colorimetric tube, and then determined by the atomic fluorescence photometer after an aqua regia (1:1) boiling water bath (99.9°C, 3 h). Meanwhile, the blank sample was prepared, serving as control. All measurements were performed in the duplicate sample (including the quality control sample of GBW07427).

### ARGs and 16S rRNA Genes in Soil Samples Detected by Real-Time Fluorescent Quantitative PCR

Total DNA was extracted from 64 soil samples using the TIANNAMP Soil DNA Kit (DP336, TIANGEN, China) according to the manufacturer’s instructions. The concentration and purity of DNA were checked using ultraviolet absorbance (Quawell Q3000, NanoDrop, America).

The 16S rRNA gene and ARGs were quantified using a StepOnePlus^™^ real-time fluorescent quantitative PCR (Thermo, United States) (Zhu et al., 2017; Zhao et al., 2018). The ARGs were quantified using a StepOnePlus^™^ real time PCR system (Wcgene Biotech, Inc., Shanghai, China). The primer sequences in the PCR reaction system and PCR amplification plots of total 28 tetracyclines, β-lactams, and sulfonamides ARGs are shown in Supplementary Table 2 and Supplementary Figure 1. The reaction mixture consisted of 5.0 μl of TB Green Premix Ex Taq II (Tli RNaseH Plus) (2×), 0.4 μl of each primer, 0.2 μl of ROX Reference Dye (50×), 1.0 μl of template DNA, and 3.0 μl of dd H_2_O. The temperature program was as follows: 30 s at 95°C, followed by 40 cycles of 5 s at 95°C, 30 s at the annealing temperature, and 72°C for 30 s. The specificity of Q-PCR products was also checked by melting curve analysis and the relative abundance of ARGs was calculated by the 2^−ΔΔCt^ method, where △Ct = Ct (target gene)—Ct (internal reference gene).

### Data Analysis

Statistical analysis was performed using Origin 2017 (OriginLab corporation), Microsoft Excel 2019 (Microsoft), and PASW Statistics 18 (IBM Information Management). The spatial distributions of heavy metals and antibiotic resistance genes in the soil of Dumont were plotted by the mapping GIS software (ArcGIS 10.4, Environmental Systems Research Institute, Inc.). Cluster 3.0 and Java Treeview 1.1.6 were used to draw a heat map to compare and analyze the differences in the selection pressure of heavy metals on ARGs. Multivariate analysis was conducted using the PCA packages in Origin 2017 to analyze the sources of heavy metals and ARGs, and then the correlations between ARGs relative abundance and heavy metal concentrations were evaluated by Cytoscape 3.6.0. All 16S rDNA sequencing data were further functionally annotated based on the KEGG database to determine the potential functions of soil microbial communities and reveal the effect of typical antibiotics on bio-enzyme activity.

## Results

### The Occurrence and Distribution of Heavy Metals in Different Soil Profiles

Eight heavy metals (i.e., As, Zn, Cu, Cr, Cd, Ni, Pb, and Hg) were detected at different depths of soil over a wide range of concentrations as represented in Table 1. It can be also found that the pH value of all the soil samples collected was higher than 8.0. All the heavy metal elements did not exceed the standard value of soil pollution risk control of agricultural land, only except for As (pH > 7.5) (GB 15618-2018). Notably, Hg were found to have a concentration below the background level in Heilongjiang Province in all soil samples. The detection illustrated the concentration of Cu and Ni in the range of 11.0–37.8 and 7.82–48.7 mg kg^−1^, respectively. As noted above, element As exhibited the greatest accumulation in the soil, accounting for a concentration (3.25–322 mg kg^−1^) which was much higher than that of other investigated elements (Qi et al., 2020). Zn had the second highest accumulation in the samples ranging from 29.8–179 mg kg^−1^, and the value was in excess of the background level by 1.17 times. In addition, the contents of Cu and Cd were also higher than the background value by 1.13 and 1.08 times, respectively. The Pearson correlation analysis of eight kinds of heavy metals showed that the content of Cu was highly correlated with Cd; Ni was highly correlated with Cr; Pb was highly correlated with As, Hg, Cr, and Cu; and Zn was highly correlated with Hg, Cd, Cr, Cu, and Pb (at the level of *p* < 0.01) (Table 2). Among the eight heavy metals, the coefficient of variation (CV) values of As was found to be higher than 0.36, indicating a severe diffusion and spatial dispersion of As in the soil by external interference.

**TABLE 1:** The statistical analysis of heavy metals in the soils.

Heavy metal	Depth/cm	Max/mg·kg^−1^	Min/mg·kg^−1^	Average ± SD/mg·kg^−1^	Background value/mg·kg^−1^	Standard/mg·kg^−1^	Single factor pollution index/*P*_*i*_	CV	Qver standard rate/%
As	10	290	3.25	182 ± 80.6	7.3	25	7.27	0.44	87.5
	50	322	6.78	219 ± 92.1	11.4		8.77	0.42	87.5
Zn	10	179	44.6	83.0 ± 36.9	70.7	300	0.277	0.44	46.9
	50	94.0	29.8	56.9 ± 17.0	69.9		0.190	0.30	25.0
Cu	10	37.8	12.4	22.6 ± 5.39	20.0	100	0.226	0.24	56.3
	50	33.0	11.0	20.0 ± 4.96	21.0		0.200	0.25	40.6
Cr	10	76.9	18.8	41.9 ± 15.4	58.6	250	0.168	0.37	15.6
	50	76.4	29.8	47.7 ± 11.5	59.5		0.191	0.24	18.8
Cd	10	0.183	0.062	0.093 ± 0.023	0.086	0.6	0.155	0.25	59.4
	50	0.113	0.043	0.074 ± 0.016	0.078		0.124	0.21	46.9
Ni	10	20.0	7.82	15.3 ± 4.11	22.8	190	0.080	0.27	0
	50	48.7	11.7	23.9 ± 7.46	24.3		0.126	0.31	31.3
Pb	10	14.0	3.86	8.29 ± 2.85	24.2	170	0.049	0.34	0
	50	27.9	6.19	13.1 ± 5.29	24.4		0.077	0.41	3.13
Hg	10	0.035	0.009	0.018 ± 0.0075	0.037	3.4	0.0052	0.42	0
	50	0.035	0.011	0.020 ± 0.0056	0.040		0.0058	0.28	0

**TABLE 2:** The correlation coefficients of heavy metal contents in the soil in the dairy farm.

	As	Hg	Cd	Cr	Cu	Ni	Pb	Zn
As	1	—	—	—	—	—	—	—
Hg	0.021	1	—	—	—	—	—	—
Cd	0.177	−0.230	1	—	—	—	—	—
Cr	0.288*	0.266*	−0.010	1	—	—	—	—
Cu	0.143	−0.284*	0.363**	−0.183	1	—	—	—
Ni	0.215	0.024	−0.180	0.335**	−0.187	1	—	—
Pb	0.409**	0.340**	−0.230	0.378**	−0.381**	0.259*	1	—
Zn	−0.142	−0.418**	0.327**	−0.394**	0.375**	-−.282*	−0.579**	1

Two asterisks indicated significant correlation (*p* < 0.01, bilateral); one asterisk indicates significant correlation (*p* < 0.05, bilateral).

Supplementary Figure 2 demonstrates the spatial distribution of the eight heavy metals in different soil layers. According to the vertical profile analysis, the pollution characteristics of As, Cr, Ni, Pb, and Hg exhibited a gradual increase from the top layer (As: 182 ± 80.6 mg kg^−1^, Cr: 41.9 ± 15.4 mg kg^−1^, Ni: 15.3 ± 4.11 mg kg^−1^, Pb: 8.29 ± 2.85 mg kg^−1^, Hg: 0.018 ± 0.0075 mg kg^−1^) to the bottom layer (As: 219 ± 92.1 mg kg^−1^, Cr: 47.7 ± 11.5 mg kg^−1^, Ni: 23.9 ± 7.46 mg kg^−1^, Pb: 13.1 ± 5.29 mg kg^−1^, Hg: 0.020 ± 0.0056 mg kg^−1^), whereas the adverse trend was observed for Cu, Zn, and Cd. According to horizontal structure analysis, Cd and Zn seemed to have similar contents in the 50 cm-deep soil layer, while a similar concentration of Cu and Zn was observed in the 10 cm-deep soil layer. Most of the heavy metals displayed an increase in concentration from the west to the east, and the most severe pollution was found in the southeastern region.

### Occurrence and Distribution of ARGs in Different Soil Layers

**FIGURE 2:**
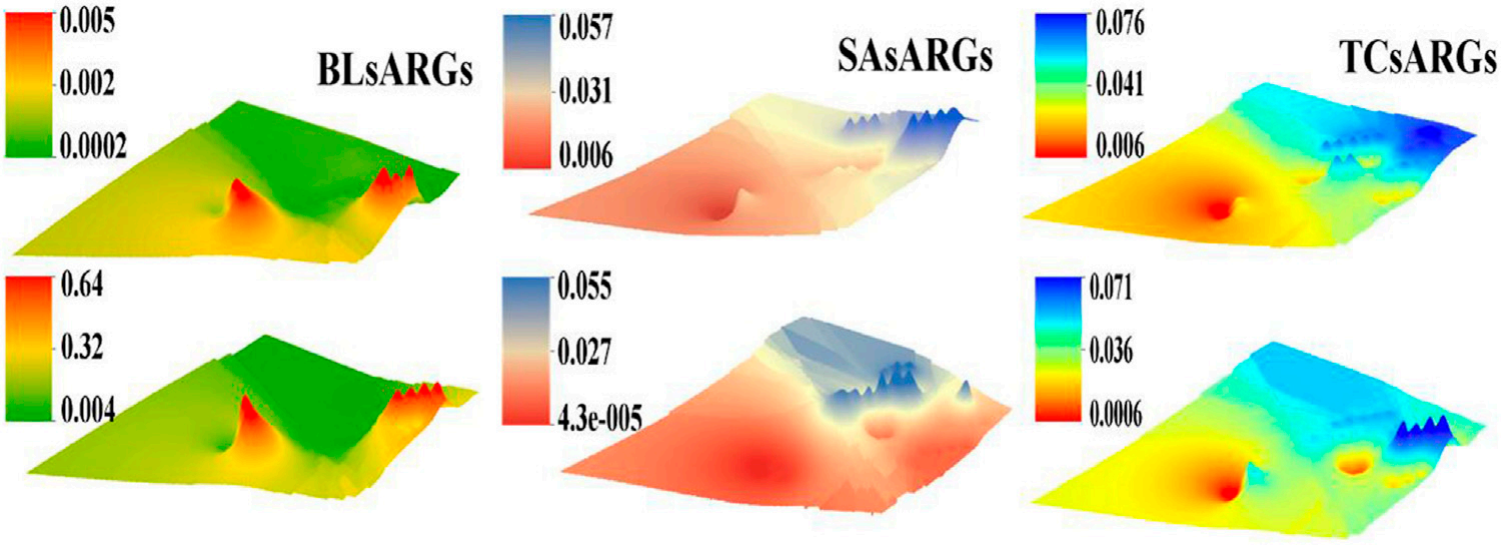
The spatial distribution of the β-lactam, sulfonamide, and tetracycline ARGs at different depths of the soil samples (top maps represent the 10 cm-deep soil and the bottom maps represent the 50 cm-deep soil).

Figure 2, Supplementary Figures 3, 4 show the detection of 28 ARGs normalized to the relative abundance based on 16S rRNA genes. In the 10 cm-deep soil layer, the relative abundances of tetracycline resistance genes (0.06–0.076) were higher than that of the β-lactam ones (0.0002–0.005) and sulfonamide ones (0.006–0.057), whereas the concentration of β-lactam (0.004–0.64) was greatly higher than that of sulfonamides (0.000043–0.055) and tetracyclines (0.0006–0.071) in the 50 cm-deep soil layer. Tetracycline resistance genes and β-lactam resistance genes were mainly concentrated in the eastern and central regions, whereas the sulfonamide resistance genes were mainly found in the eastern area of the dairy farm. The comparison of the ARG relative abundance reflected that the east and central zones of the area were the most seriously contaminated among the investigated regions. The soil samples collected near both the west and north areas contained the lowest level of ARG relative abundance (0.001). All ARGs tended to decline gradually with the increase of the soil depth except for the β-lactam ARGs.

### The Effect of Typical Antibiotics on Bioactivity Activity

The relative abundances of tetracycline resistance genes were the highest among the three categories at 0.06–0.076 in the 10 cm-deep soil layer and the relative abundances of β-lactam resistance genes were the highest at 0.004–0.64 in the 50 cm-deep soil layer. It was clear that tetracycline antibiotics (TCs) in the 10 cm-deep soil layer and the β-lactam antibiotics (BLs) in the 50 cm-deep soil layer were the main antibiotic categories. A previous study reported that tetracyclines had a strong inhibitory effect on the biosynthesis of proteins and nucleic acids of microbes, which contributed considerably to ARG formation (Roberts, 2019). Based on the results of the functional annotation of 16S rDNA fragments based on the KEGG database, it is clear that antibiotics have a significant inhibitory effect on multiple metabolism processes, such as metabolism, transport and catabolism, and membrane transport. Most bio-enzymes were found to participate in low activities in different pathways. For example, most bio-enzymes that participated in the cysteine and methionine metabolism (Supplementary Figure 5) were found to be downregulated, which was greatly inhibited owing to the presence of antibiotics in soil.

### Principal Component Analysis for Heavy Metals and Antibiotic Resistance Genes

**FIGURE 3:**
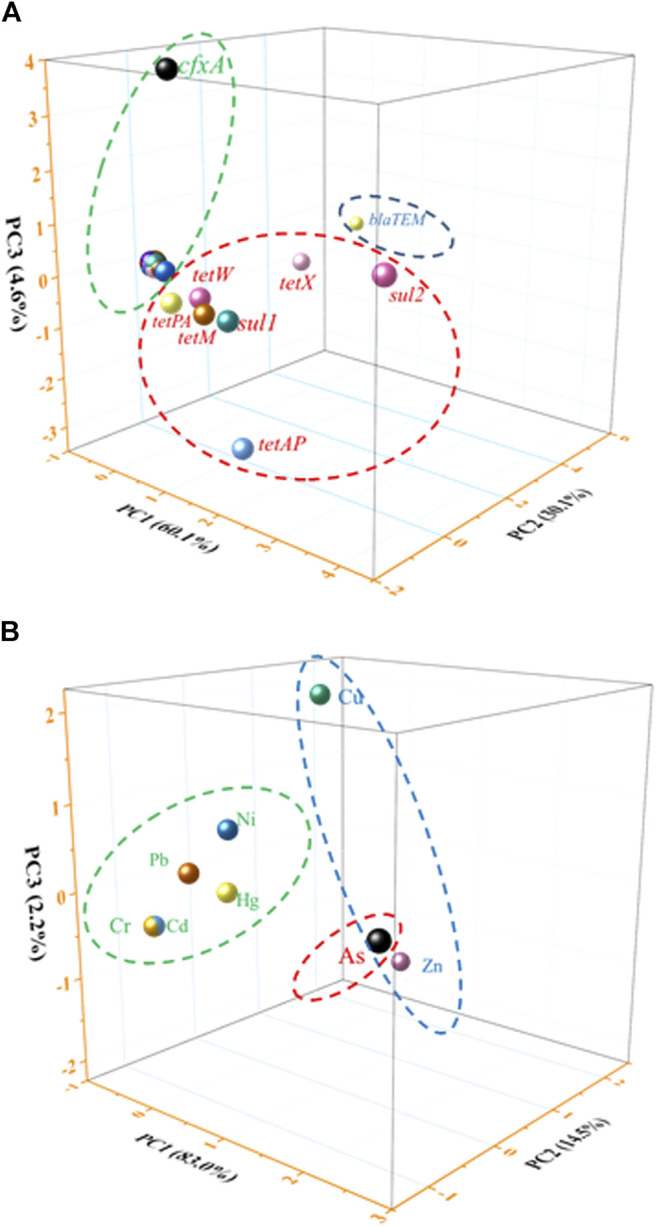
The PCA analysis of the ARGs and heavy metals in the soil. **(A)** ARGs results and **(B)** heavy metals results.

The source of heavy metals and ARGs was calculated by principal component analysis (PCA) (Figure 3 and Supplementary Table 3). The first three principal components explained 60.1, 30.1, and 4.6% of the total ARG variations in all samples, respectively. Then 28 kinds of ARGs were selected to make the principal component scores according to the correspondent factor scores multiplied by the arithmetic square root of corresponding variance, which yielded the comprehensive pollution index. Pearson correlation analysis of the total 28 kinds of ARGs were listed (Supplementary Table 4), it indicated significant correlation (*p* < 0.01). Table 3 summarizes the relative abundance of ARGs in the soil samples, which followed the orders of *sul2* > *tetX* > *blaTEM* > *tetAP* > *sul1* > *tetM* > *tetW* > *cfxA* > *tetPA* > *tetZ* > *tetG* > *sul3* > *tetT* > *tetS* > *blaOXA1* > *tetY* > *tetC* > *tetO* > *tetB* > *fox5* > *tet36* > *tet34* > *blaPSE* > *tet32* > *ampC2* > *tetR* > *blaOXA10* > *ampC4*.

**TABLE 3:** The comprehensive score of the PCA analysis for the ARGs.

ARGs	FAC1	FAC2	FAC3	FAC4	F1	F2	F3	F4	Comprehensive score	Comprehensive ranking
*sul2*	3.721	−0.365	0.935	−1.001	23.1	−1.60	1.60	−1.28	13.8	1
*tetX*	2.329	−0.234	0.774	1.927	14.4	−1.03	1.33	2.47	8.72	2
*blaTEM*	0.208	5.091	0.009	−0.003	1.29	22.3	0.016	−0.003	7.70	3
*tetAP*	1.293	−0.317	−3.077	2.480	8.02	−1.39	−5.28	3.18	4.35	4
*sul1*	1.035	−0.276	−0.672	−3.546	6.42	−1.21	−1.15	−4.55	3.41	5
*tetM*	0.484	−0.143	−0.733	−1.168	3.00	−0.629	−1.26	−1.50	1.56	6
*tetW*	0.322	−0.019	−0.491	0.291	2.00	−0.084	−0.842	0.373	1.18	7
*cfxA*	−0.021	−0.244	3.789	1.139	−0.13	−1.07	6.50	1.46	−0.06	8
*tetPA*	−0.112	−0.216	−0.666	0.549	−0.69	−0.948	−1.14	0.704	−0.76	9
*tetZ*	−0.245	−0.186	−0.077	0.019	−1.52	−0.816	−0.133	0.024	−1.19	10
*tetG*	−0.390	−0.187	−0.036	−0.141	−2.42	−0.819	−0.062	−0.181	−1.75	11
*sul3*	−0.428	−0.205	−0.052	−0.645	−2.66	−0.901	−0.089	−0.826	−1.94	12
*tetT*	−0.442	−0.199	0.030	−0.212	−2.74	−0.871	0.051	−0.271	−1.97	13
*tetS*	−0.491	−0.088	0.012	−0.029	−3.04	−0.388	0.021	−0.037	−2.00	14
*blaOXA1*	−0.506	−0.101	0.043	0.023	−3.14	−0.443	0.074	0.030	−2.07	15
*tetY*	−0.498	−0.167	0.020	0.021	−3.09	−0.734	0.033	0.026	−2.13	16
*tetC*	−0.520	−0.116	0.011	0.017	−3.22	−0.511	0.019	0.022	−2.15	17
*tetO*	−0.501	−0.188	0.034	0.001	−3.10	−0.823	0.059	0.002	−2.17	18
*tetB*	−0.520	−0.147	0.011	0.046	−3.22	−0.645	0.019	0.058	−2.19	19
*fox5*	−0.514	−0.178	0.020	−0.040	−3.19	−0.783	0.034	−0.052	−2.21	20
*tet36*	−0.513	−0.195	0.035	0.064	−3.18	−0.854	0.060	0.082	−2.22	21
*tet34*	−0.517	−0.195	−0.037	0.133	−3.21	−0.855	−0.064	0.171	−2.24	22
*blaPSE*	−0.522	−0.190	0.031	0.006	−3.24	−0.835	0.053	0.007	−2.25	23
*tet32*	−0.523	−0.192	0.047	-0.003	−3.24	−0.840	0.080	−0.004	−2.26	24
*ampC2*	−0.529	−0.174	0.008	0.021	−3.28	−0.765	0.014	0.027	−2.26	25
*tetR*	−0.531	−0.190	0.007	0.013	−3.29	−0.832	0.012	0.017	−2.29	26
*blaOXA10*	−0.532	−0.189	0.015	0.019	−3.30	−0.830	0.026	0.024	−2.29	27
*ampC4*	−0.536	−0.190	0.010	0.020	−3.32	−0.834	0.017	0.025	−2.31	28

For different depths of soil samples, three major groups of data accounted for 99.7% of the total variance, which played a vital role in tracing the sources of contamination by heavy metals in the dairy farm. Considering the same source for all detected heavy metals (Figure 3 and Table 4), we attributed the natural source to be the most likely reason responsible for the detection of heavy metals in the soil (Hg, Cd, Cr, Ni, and Pb), except for Zn and Cu which may have come from the feed addition, and As due to its severe contamination.

**TABLE 4:** Total PCA variance of heavy metal contents in soil samples.

Principal component number	Initial eigenvalue λ			Extraction sums of squared loadings		
	Total	Percent of variance (%)	Cumulative (%)	Total	Percent of variance (%)	Cumulative (%)
1	53.1	83.0	83.0	53.1	83.0	83.0
2	9.30	14.5	97.5	9.30	14.5	97.5
3	1.42	2.21	99.7	1.42	2.21	99.7
4	0.101	0.158	99.9	—	—	—
5	0.058	0.090	100	—	—	—
6	0.027	0.042	100	—	—	—
7	3.37E-07	5.26E-07	100	—	—	—
8	7.39E-31	1.15E-30	100	—	—	—

### Correlation Between Heavy Metals and Antibiotic Resistance Genes

**FIGURE 4:**
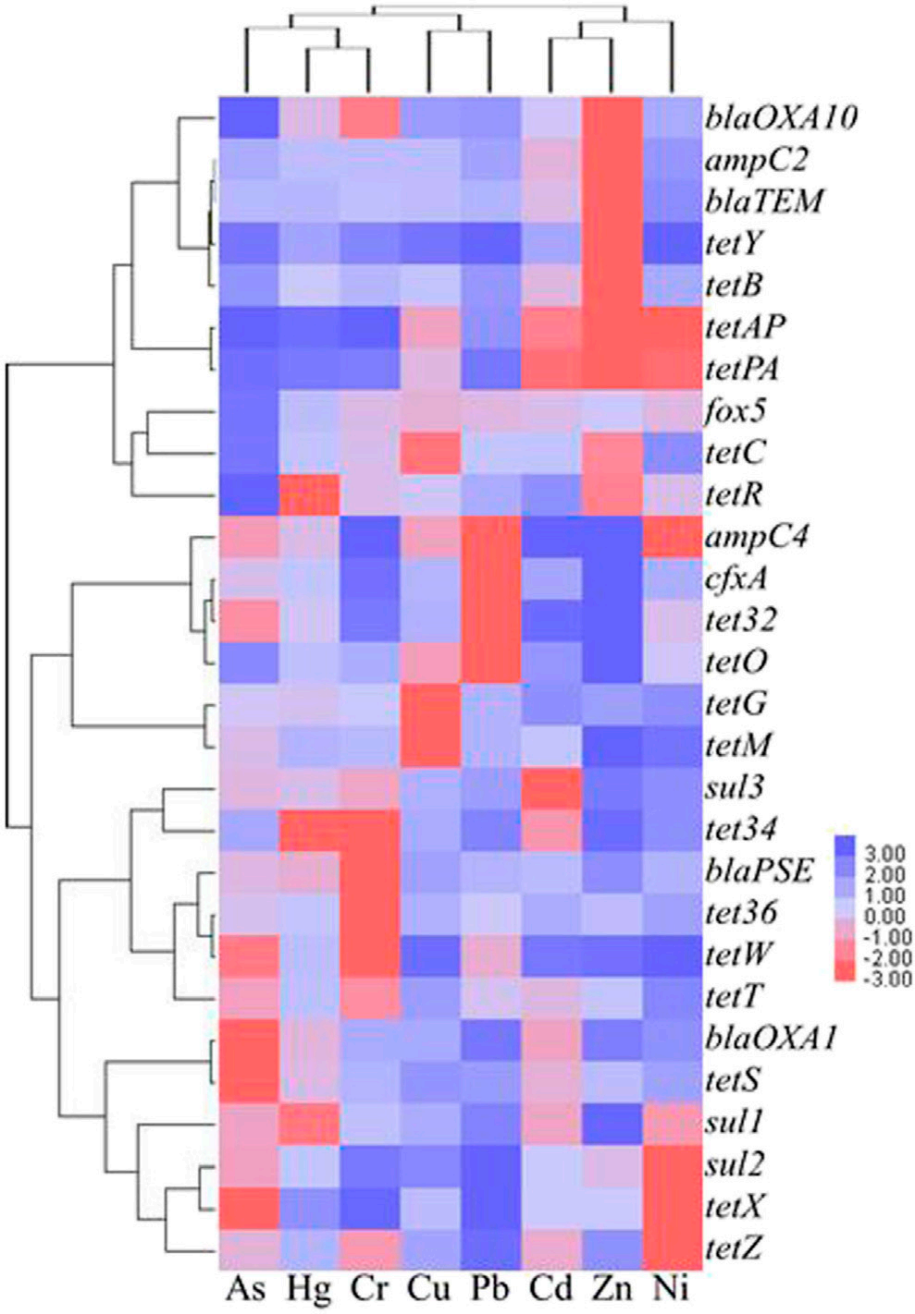
The clustering analysis of the heavy metals and the ARGs within the 64 soil samples drawn using the Java Treeview software.

**FIGURE 5:**
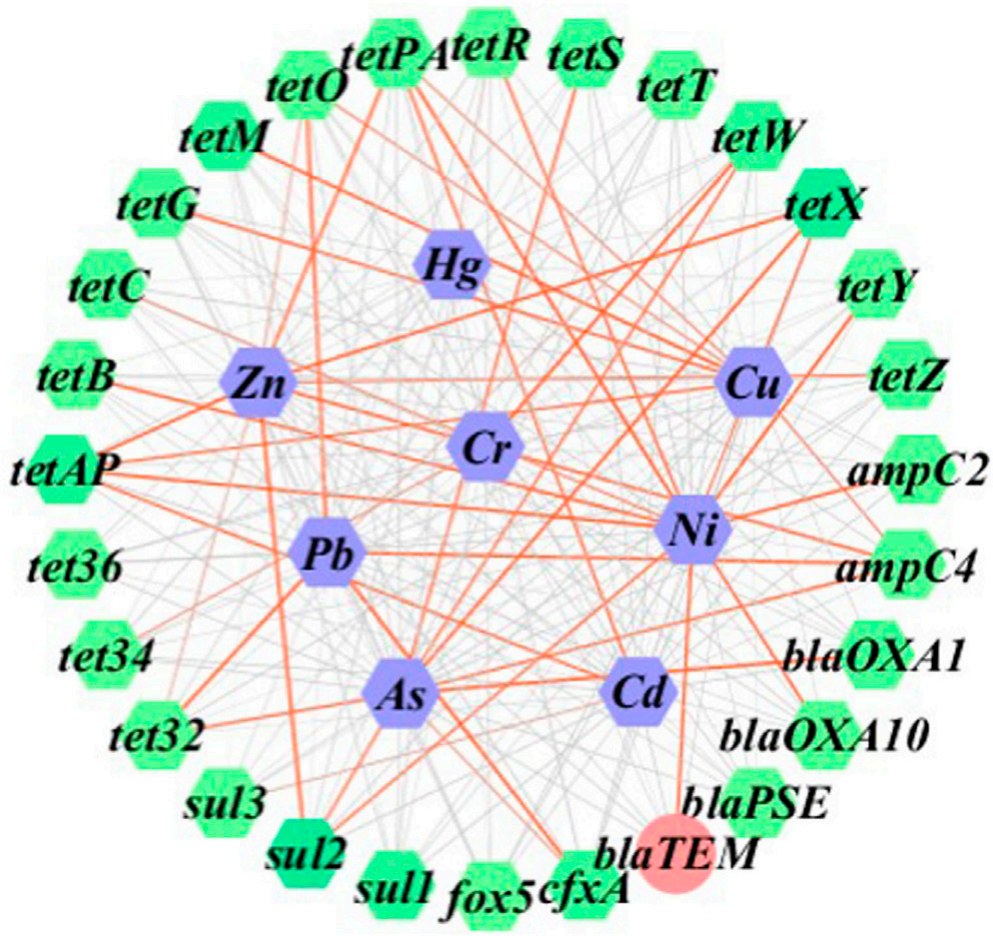
Gene network image showing the correlations between ARGs and heavy metal elements across the 64 soil samples in total.

Table 5 shows the analytic results of correlation between heavy metals and ARGs. Based on the level of *p* < 0.05, a low relevance was observed for As and *sul2* (*r* = 0.314), *tet32* (*r* = 0.314), and *tetW* (*r* = 0.317), the case was quite similar to that for Ni and *sul2* (*r* = -0.317). In comparison, Ni demonstrated a moderate correlation with *tetY* at the level of *p* < 0.01 (*r* = 0.576). Based on the level of *p* < 0.01, a low relevance was observed for Cr and *tetW* (*r* = 0.413), Cu and *tetM* (*r* = 0.415), Ni and *tetPA* (*r* = -0.413), *and tetAP* (*r* = -0.439), the result being quite similar to that for Pb and *ampC4* (*r* = 0.425), *cfxA* (*r* = 0.481), *tet32* (*r* = 0.466), *tetO* (*r* = 0.477), Zn and *sul2* (*r* = 0.450), *tetAP* (*r* = 0.415), and *tetX* (*r* = 0.400). A low relevance on the level of *p* < 0.01 was also observed for As and *ampC4* (*r* = 0.326), *blaOXA1* (*r* = -0.399), *tetS* (*r* = -0.322), *tetX* (*r* = 0.377), Cd and *tetAP* (*r* = 0.377), *tetPA* (*r* = 0.332), Cu and *tetAP* (*r* = 0.319), *tetG* (*r* = 0.337), Ni and *ampC2* (*r* = 0.356), *blaOXA10* (*r* = 0.353), *blaTEM* (*r* = 0.353), *tetB* (*r* = 0.355), *tetR* (*r* = 0.325), *tetX* (*r* = -0.300), Zn and *ampC4* (*r* = -0.377), *tetPA* (*r* = 0.364), and *tetZ* (*r* = 0.302). Hg was shown to be insignificantly correlated with the formation of ARGs in this study. According to cluster analysis shown in Figure 4, the heatmaps of transverse reflect the relative abundance of ARGs, the longitudinal represents the clustering situation of eight kinds of heavy metal and the reaction of heavy metals to the ARGs similarity selection pressure. Zn, Cd, and Ni can cluster together, and Cr, Hg, and As with Pb and Cu can be grouped. This indicated the similar selective pressure of Ni and Cd, Zn for ARGs formation, and likewise the similar selective pressures of other types of heavy metals except for Cd, Zn, and Ni. The co-occurrence pattern between heavy metals and ARGs explored by using network analysis based on Pearson correlation (Figure 5) agreed well with the cluster analysis on selective pressure effects of As, Ni, Cu, Pb, and Zn on ARG formation.

**TABLE 5:** The correlations between heavy metal content and relative abundances of the ARGs.

	As	Hg	Cd	Cr	Cu	Ni	Pb	Zn
*ampC2*	−0.148	0.033	−0.162	−0.066	0.118	**0.356**	−0.112	−0.099
	0.243	0.795	0.200	0.602	0.353	**0.004**	0.380	0.434
*ampC4*	**0.326**	0.220	−0.063	0.000	−0.290	0.050	**0.425**	−**0.377**
	**0.009**	0.080	0.622	0.999	**0.020**	0.693	**0.000**	**0.002**
*blaOXA1*	−**0.399**	0.129	−0.199	−0.049	−0.103	0.143	−0.043	−0.111
	**0.001**	0.311	0.116	0.701	0.418	0.259	0.738	0.383
*blaOXA10*	−0.002	−0.104	−0.134	−0.214	0.058	**0.353**	−0.092	0.137
	0.985	0.415	0.290	0.089	0.651	**0.004**	0.468	0.282
*blaPSE*	0.189	−0.085	−0.050	−0.231	−0.008	0.117	0.104	0.112
	0.134	0.503	0.695	0.066	0.951	0.359	0.414	0.377
*blaTEM*	−0.172	0.024	−0.155	−0.082	0.126	**0.353**	−0.145	−0.088
	0.175	0.852	0.220	0.518	0.322	**0.004**	0.254	0.488
*cfxA*	0.272	0.160	−0.156	−0.026	−0.194	0.067	**0.481**	−0.220
	**0.030**	0.207	0.217	0.836	0.125	0.596	**0.000**	0.081
*fox5*	−0.033	0.024	−0.143	−0.121	−0.177	0.229	0.197	−0.204
	0.795	0.848	0.261	0.342	0.163	0.068	0.119	0.106
*sul1*	0.240	−0.201	0.183	0.076	−0.089	−0.085	−0.044	0.243
	0.057	0.111	0.147	0.552	0.486	0.503	0.728	0.053
*sul2*	**0.314**	−0.183	0.226	−0.084	0.154	−**0.317**	−0.026	**0.450**
	**0.011**	0.147	0.073	0.508	0.223	**0.011**	0.839	**0.000**
*sul3*	0.182	−0.030	0.264	−0.110	0.029	−0.063	−0.038	0.012
	0.150	0.815	**0.035**	0.387	0.818	0.619	0.764	0.928
*tet32*	**0.314**	0.176	−0.054	−0.046	−0.188	0.030	**0.466**	−**0.265**
	**0.012**	0.163	0.671	0.715	0.137	0.812	**0.000**	**0.035**
*tet34*	0.136	0.218	0.196	0.274	0.079	−0.089	0.037	−0.077
	0.282	0.083	0.120	**0.028**	0.534	0.486	0.770	0.543
*tet36*	0.202	−0.041	0.054	−0.242	0.087	−0.210	0.172	0.113
	0.110	0.747	0.672	0.054	0.492	0.095	0.173	0.376
*tetAP*	0.160	−0.120	**0.337**	0.087	**0.319**	−**0.439**	−0.249	**0.415**
	0.207	0.344	**0.007**	0.493	**0.010**	**0.000**	**0.048**	**0.001**
*tetB*	−0.098	0.053	−0.146	−0.033	0.117	**0.355**	−0.075	−0.121
	0.442	0.678	0.250	0.798	0.357	**0.004**	0.558	0.341
*tetC*	−0.026	−0.026	−0.095	−0.106	0.242	0.289	−0.155	−0.062
	0.840	0.838	0.457	0.404	0.055	**0.020**	0.222	0.625
*tetG*	0.206	−0.098	0.005	−0.105	**0.337**	0.185	−0.147	0.097
	0.103	0.439	0.969	0.408	**0.007**	0.144	0.247	0.447
*tetM*	0.229	−0.045	0.139	−0.089	**0.415**	−0.031	−0.159	0.057
	0.069	0.726	0.273	0.482	**0.001**	0.810	0.209	0.655
*tetO*	0.203	0.174	−0.157	0.168	−0.283	0.017	**0.477**	−0.270
	0.107	0.168	0.216	0.185	**0.023**	0.897	**0.000**	**0.031**
*tetPA*	0.201	−0.098	**0.332**	0.129	0.281	−**0.413**	−0.192	**0.364**
	0.111	0.439	**0.007**	0.308	**0.024**	**0.001**	0.128	**0.003**
*tetR*	−0.009	−0.254	−0.060	−0.162	0.187	**0.325**	−0.167	0.231
	0.944	**0.043**	0.639	0.201	0.140	**0.009**	0.188	0.066
*tetS*	−**0.322**	0.083	−0.153	−0.019	−0.008	0.201	−0.077	−0.102
	**0.009**	0.514	0.228	0.881	0.952	0.111	0.547	0.422
*tetT*	0.246	−0.049	0.160	−0.194	−0.063	−0.226	0.190	0.077
	0.050	0.701	0.207	0.124	0.623	0.073	0.133	0.543
*tetW*	**0.317**	0.120	0.034	**0.413**	−0.041	−0.168	0.257	−0.063
	**0.011**	0.343	0.788	**0.001**	0.749	0.184	**0.040**	0.622
*tetX*	**0.377**	−0.089	0.222	−0.030	0.215	−**0.300**	0.058	**0.400**
	**0.002**	0.485	0.078	0.813	0.088	**0.016**	0.650	**0.001**
*tetY*	0.121	−0.061	−0.123	0.022	−0.045	**0.576**	−0.063	−0.045
	0.341	0.633	0.333	0.863	0.725	**0.000**	0.618	0.726
*tetZ*	0.229	−0.020	0.182	0.186	0.083	−0.164	−0.004	**0.302**
	0.068	0.877	0.150	0.140	0.516	0.196	0.975	**0.015**

Note: In each cell, the top value indicated the Pearson correlation coefficient (*r*), and the bottom value in italics indicated the *p*-value. Bold values indicated statistical significance (*|r|*≥0.3 *or p*＜0.05).

## Discussion

Soil is a major source of metal, and at the same time it also acts as a natural barrier that prevents the food chain from being contaminated by pollutants and toxins. However, the excessive accumulation of heavy metals leads to a decline in quality of the soil environment, which is the main consequence of the transportation of heavy metals into soil mediums in a variety of pathways. Thus, increased attention needs to be paid to the accumulation of heavy metals in agricultural products and their potential impacts to human health through the food chain (Bi et al., 2018; Han et al., 2018). This is a matter of particular importance for dairy farms so as to provide a commercially safe and reliable supply of agricultural products like milk and beef. Our results clearly indicated severe As contamination whose concentration increased with the vertical depth of the soil layer. Atmospheric subsidence and the application of pesticides and herbicides may constitute the most likely source of As in the dairy farm (Yao et al., 2019). Taking account of the relatively low average content of Hg in this area, it seems that the distribution characteristics of Hg are in relation to the natural origin of mother rock and soil (Hou et al., 2019). On the contrary, Cu, Zn, and Cd could be enriched more easily in the surface layer of the soil profile. The results obtained agreed well with that reported by Liang et al. (2018), Zhang et al. (2019a). It was noteworthy that the atmospheric deposition of Cd, Cu, and Zn might contribute to heavy pollution (Chen et al., 2018). Most of the heavy metals can not only accumulate easily on the surface soil, but also migrate with the increased soil depth (Li et al., 2017a). Within this context, the correlation analysis among different types of heavy metals clearly showed a positive correlation between Ni and Cr, and Zn and Cu in the soil (*p* < 0.01), reflected by correlation coefficients greater than 30%. This suggested the fact that Ni and Cr, Zn and Cu had the same resources and hid the risks of combined pollution. In comparison, there was a slight correlation between Hg and Cd, indicating the different behavior of pollution source and transportation.

With the constantly emerging environmental issues, ARGs may lead to more severe environmental and ecological risks than antibiotics in the soil. For different depths of soil layers, all the ARGs examined were mainly concentrated in the east region of the area (T1-T32). The comparative analysis of relative abundance for ARGs showed that the east and central zones were contaminated more severely than other areas, preliminarily due to more activities of adult cows in free regions. The lowest relative abundance of ARGs found near the west and south areas of the dairy farm appeared to be similar due to the lack of human activities with the calves. The transfer of ARGs can be affected by several factors including, but not limited to, physical and chemical properties of the soil, antibiotics, heavy metals, and bacterial communities. Moreover, the wide usage of antibiotics can further lead to the continuous accumulation of ARGs. With the increased depth of the soil layer, the anoxic conditions will be unfavorable for the growth of aerobic microorganisms, and thus the content of ARGs tends to be declined owing to the inhibition effect of oxygen to the activity of resistant microbes. Hence, these results indicated that ARGs had a strong ability of movement transfer and a wide range of activity. Tetracycline, sulfonamide, and β-lactam resistance genes were the most commonly identified ARGs in soils. Among the 17 tetracycline resistance genes, all ARGs except *tet32, tet34, tet36, tetPA,* and *tetR* were detected in the soil samples. However, it seems interesting to find a lower concentration of *tetR* than other ARGs in some samples, possibly due to the use of penicillin or tetracycline on dairy farms to prevent cow mastitis caused by *Staphylococcus aureus, Streptococcus, Mycoplasma, and virus* (Zhang, 2017). It is worth noting that oxytetracycline-rich and oxytetracycline-free manure equally enriches soil tetracycline resistome (Kyselková et al., 2013). For sulfonamide resistance genes, *sul1* and *sul2* had the highest relative abundance among all the samples. The persistent exposure to sulfonamides tended to impose selective pressure for *sul1* and *sul2* resistance genes in soils, and hence sulfamethoxazole contamination can considerably increase the relative abundance of sulfamethoxazole resistance genes *sul1* and *sul2* (Zhang et al., 2019b). The results observed in this study were shown to agree well with those reported in recent surveys conducted in Northern China (Zhang et al., 2016), where *sul1* and *sul2* were 100% detectable. The content of β-lactam resistance genes displayed an obvious upward tendency with the increase in soil depth, especially in the areas of T9-T14, T17-T21, and T29-T32. This suggested that the risk remained for not only downward vertical transmission of β-lactam ARGs, but also transmission between parent and offspring in cows. This implied a higher usage and release of β-lactams in the dairy farm under the pressure of cephalosporins or heavy metals (Li et al., 2017b). S*ul2*, *tetX*, *and blaTEM* were universally distributed with high abundance in different depths of soils in the dairy farm, indicating the requirement for paying more attention to these genes in future. It is also possible that the different distribution of ARGs may have connections with the prevalence of resistant genes and mode of antibiotic application in different regions.

Theoretically, the ARGs are supposed to disappear in the absence of antibiotic selection pressure. However, the widespread spread of ARGs and long presence in human pathogens in the areas without high antibiotic loading showed that once tetracycline, sulfonamide, and β-lactam resistance genes are present in the platforms available for gene transfer, they would be likely to sustain for a long period of time in natural ecosystems. When the ARGs existing in the environment are polluted by heavy metals or fungicides, the co-selection of metal and mobile genetic elements (MGEs) can also trigger horizontal transfer of ARGs (Von Wintersdorff et al., 2016; Imran et al., 2019). In fact, the heavy metals may be more selective to ARGs than specific antibiotics in some cases (Ji et al., 2012), indicating some essential correlations between heavy metals and the formation of ARGs as evidenced in previous studies (Guo et al., 2018; Qiao et al., 2018; Zhao et al., 2019a; Dickinson et al., 2019). To further elucidate the relationships in dairy farmland, we performed network analysis on the experimental data representing the samples collected from all points including 28 ARGs and eight heavy metals. The significant correlations between ARGs and heavy metals clearly suggested a crucial role played by heavy metal contamination in observation of ARG relative abundance. In a case study carried out in Northern Ireland by Zhao et al. (2019b), there were positive correlations between 24 types of metals and ARGs, particularly for that of Cr, Zn, and β-lactam ARGs. Regarding the influence of heavy metals, antibiotics, and nutrients on ARGs in fertilized soil, Deng et al. (2019) demonstrated the notable dependence of ARG formation on copper elements. Likewise, similar conclusions were also be drawn by Knapp et al. (2011) who reported the selection pressure induced by Cu, Cr, Ni, Pb, and Fe to the content of ARGs in Scottish soil. Ding et al. (2019) proved to be a strong synergistic effect on ARGs when Zn or Cu co-existed together with oxytetracycline. In our study, the abundance of ARGs detected was shown to be in close correlation with As, Cu, Ni, Pb, and Zn. Therefore, although heavy metals have a potential impact on ARGs in soils, the combination of multiple factors is often the reason for the difference in the selection of ARGs by heavy metals. The mechanisms of synergistic selection resistance of the heavy metals and ARGs should be associated with the processes where synergistic resistance, cross resistance, synergistic regulation, and biofilm-formation induction happens (Baker-Austin et al., 2006). As we mentioned before, ARGs are gene fragments that allow microorganisms to grow and reproduce in response to exposure to antibiotics, their transmission is relayed by MGEs, the expression of MGEs is effected by heavy metals and lead to ARG abundance; heavy metals in the soil put co-selective stress on ARGs by co-resistance, cross-resistance, and co-regulation by occupying MGEs. The dairy farm had saline-alkali soil with a pH value higher than 7.0, a key factor that had an important impact on the behavior and bioavailability of heavy metals. The heavy metals and ARGs may have the same space-occupying MGEs, and the transcription and translation response systems of microorganisms in soil.

Taken together, the results of this study provides a deep mechanistic insight into the diversity of ARGs and response to heavy metals in the soils of dairy farms. We herein showed that heavy metal contamination in the soil can promote the co-regulation of the expression of ARGs. The visual and deep insight into the correlation between the distribution of typical heavy metals and ARGs by the GIS technique will be of great significance to provide useful information for soil remediation of dairy farms.

## Conclusion

In this study, heavy metals and ARGs were demonstrated to co-exist which showed significant diversity in concentrations in soils of a dairy farm. Heavy metal As showed a top-down vertical pollution, while Cu, Zn, and Cd seemed more likely to be enriched at the surface. These phenomena reflected the span of evolution. Typically, heavy metals (As, Cu, Ni, Pb, and Zn) played an important role in the influence of the abundance of certain ARGs, but the combination of multiple factors was often the reason for the difference in the selection of ARGs by heavy metals, such as: antibiotics, heavy metals, the physical and chemical properties, bacterial community structure, and so on. In addition, most of the heavy metals were high in concentration, but ARGs were lower apart from *blaTEM.* The amount of pollution by these elements was higher in the soils since the dairy farm utilized supplement feed, antibiotics, and other agricultural production. According to the functional annotations, antibiotics have a significant inhibitory effect on various metabolism process. Most bio-enzymes were found to have low activities in different pathways. More actions should be taken to lessen the possibilities of ARGs and heavy metals entering into the human food chain through cow’s milk and beef. This study gives a visual and deep insight into correlations between distribution of typical heavy metals and ARGs using the GIS technique, which can provide guidance for future development of enzyme-based smart materials.

## Data Availability

The original contributions presented in the study are included in the article/Supplementary Material, further inquiries can be directed to the corresponding authors.
