# Supplementary material for: The Interactions Between Antibiotic Resistance Genes and Heavy Metal Pollution Under Co-Selective Pressure Influenced the Bio-Enzyme Activity
Source: Front Chem. 2021 Jul 14;9:691565. doi: 10.3389/fchem.2021.691565 (PMC8316601; doi:10.3389/fchem.2021.691565)
Supplement: Supplementary file 1 [file DataSheet3.DOCX]

TABLE S1 Latitude and longitude of on-site sampling points designed by GIS

| Sample number  （10-cm/50-cm-depth soil） | Longitude and latitude of location |
| --- | --- |
| T01/T33 | 46.846804°N, 124.604584°E |
| T02/T34 | 46.846804°N, 124.604447°E |
| T03/T35 | 46.846804°N, 124.604310°E |
| T04/T36 | 46.846667°N, 124.604584°E |
| T05/T37 | 46.846667°N, 124.604447°E |
| T06/T38 | 46.846667°N, 124.604310°E |
| T07/T39 | 46.846667°N, 124.604163°E |
| T08/T40 | 46.846667°N, 124.604027°E |
| T09/T41 | 46.846531°N, 124.604447°E |
| T10/T42 | 46.846531°N, 124.604310°E |
| T11/T43 | 46.846531°N, 124.604163°E |
| T12/T44 | 46.846531°N, 124.604027°E |
| T13/T45 | 46.846531°N, 124.603890°E |
| T14/T46 | 46.846531°N, 124.603753°E |
| T15/T47 | 46.846394°N, 124.604163°E |
| T16/T48 | 46.846394°N, 124.604027°E |
| T17/T49 | 46.846394°N, 124.605004°E |
| T18/T50 | 46.846257°N, 124.605141°E |
| T19/T51 | 46.846257°N, 124.605004°E |
| T20/T52 | 46.846257°N, 124.604857°E |
| T21/T53 | 46.846257°N, 124.604720°E |
| T22/T54 | 46.846110°N, 124.604857°E |
| T23/T55 | 46.846110°N, 124.604720°E |
| T24/T56 | 46.846110°N, 124.604584°E |
| T25/T57 | 46.846110°N, 124.604447°E |
| T26/T58 | 46.845974°N, 124.604584°E |
| T27/T59 | 46.845974°N, 124.604447°E |
| T28/T60 | 46.845974°N, 124.604310°E |
| T29/T61 | 46.846394°N, 124.603059°E |
| T30/T62 | 46.846257°N, 124.603059°E |
| T31/T63 | 46.846531°N, 124.602923°E |
| T32/T64 | 46.846394°N, 124.602923°E |

TABLE S2 Primer sequences in PCR reaction system

| Gene | Forward prime | Reversed prime |
| --- | --- | --- |
| *ampC2* | GCAGCACGCCCCGTAA | TGTACCCATGATGCGCGTACT |
| *ampC4* | TCCGGTGACGCGACAGA | CAGCACGCCGGTGAAAGT |
| *blaOXA1/blaOXA30* | CGGATGGTTTGAAGGGTTTATTAT | TCTTGGCTTTTATGCTTGATGTTAA |
| *blaOXA10* | CGCAATTATCGGCCTAGAAACT | TTGGCTTTCCGTCCCATTT |
| *blaPSE* | TTGTGACCTATTCCCCTGTAATAGAA | TGCGAAGCACGCATCATC |
| *blaTEM* | AGCATCTTACGGATGGCATGA | TCCTCCGATCGTTGTCAGAAGT |
| *cfxA* | TCATTCCTCGTTCAAGTTTTCAGA | TGCAGCACCAAGAGGAGATGT |
| *fox5* | GGTTTGCCGCTGCAGTTC | GCGGCCAGGTGACCAA |
| *sul1* | CACCGGAAACATCGCTGCA | AAGTTCCGCCGCAAGGCT |
| *sul2* | GTCAAAGAACGCCGCAATGT | TCATCTGCCAAACTCGTCGTTA |
| *sul3* | TCCGTTCAGCGAATTGGTGCAG | TTCGTTCACGCCTTACACCAGC |
| *tet32* | CCATTACTTCGGACAACGGTAGA | CAATCTCTGTGAGGGCATTTAACA |
| *tet34* | CTTAGCGCAAACAGCAATCAGT | CGGTGATACAGCGCGTAAACT |
| *tet 36* | AGAATACTCAGCAGAGGTCAGTTCCT | TGGTAGGTCGATAACCCGAAAAT |
| *tetAP* | AGTTGCAGATGTGTATAGTCGTAAACTATCTATT | TGCTACAAGTACGAAAACAAAACTAGAA |
| *tetB* | GCCCAGTGCTGTTGTTGTCAT | TGAAAGCAAACGGCCTAAATACA |
| *tetC* | ACTGGTAAGGTAAACGCCATTGTC | ATGCATAAACCAGCCATTGAGTAAG |
| *tetG* | TCAACCATTGCCGATTCGA | TGGCCCGGCAATCATG |
| *tetO* | ATGTGGATACTACAACGCATGAGATT | TGCCTCCACATGATATTTTTCCT |
| *tetR* | CGCGATAGACGCCTTCGA | TCCTGACAACGAGCCTCCTT |
| *tetS* | TTAAGGACAAACTTTCTGACGACATC | TGTCTCCCATTGTTCTGGTTCA |
| *tetT* | CCATATAGAGGTTCCACCAAATCC | TGACCCTATTGGTAGTGGTTCTATTG |
| *tetW* | ATGAACATTCCCACCGTTATCTTT | ATATCGGCGGAGAGCTTATCC |
| *tetX* | AAATTTGTTACCGACACGGAAGTT | CATAGCTGAAAAAATCCAGGACAGTT |
| *tetY* | ATTTGTACCGGCAGAGCAAAC | GGCGCTGCCGCCATTATGC |
| *tetZ* | CCTTCTCGACCAGGTCGG | ACCCACAGCGTGTCCGTC |


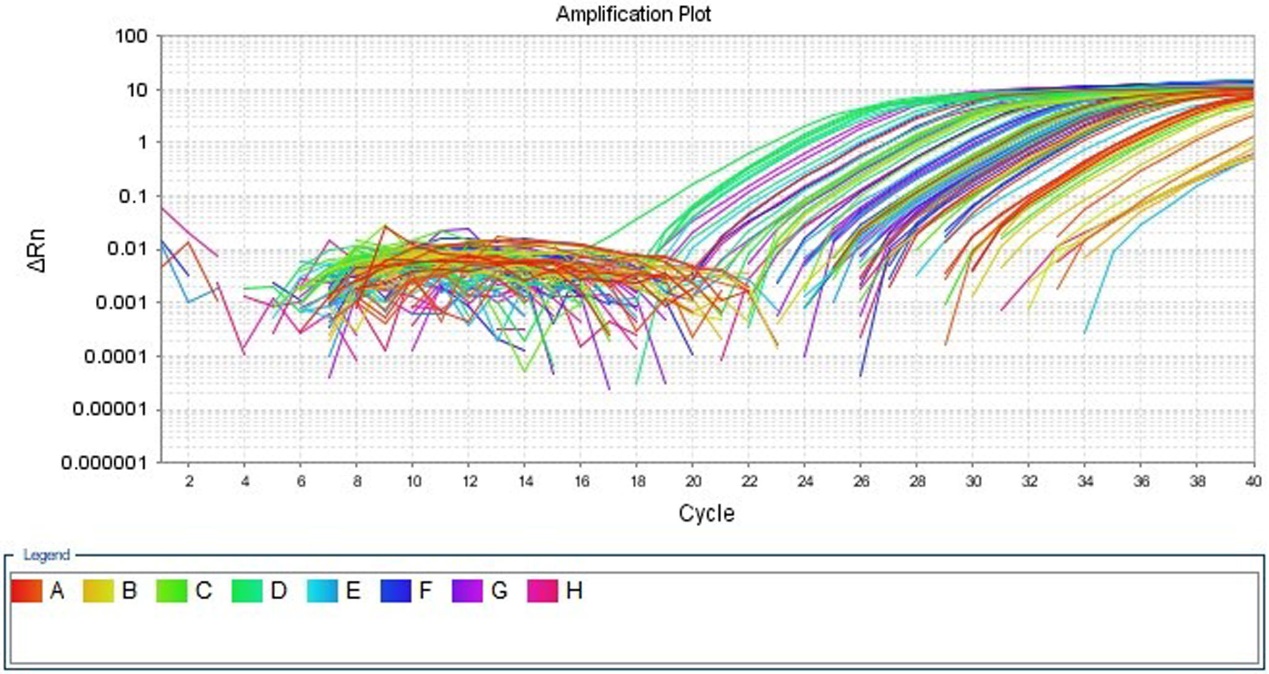


**FIGURE S1** The PCR amplification plots of the total 28 tetracyclines, β-lactams and sulfonamides ARGs


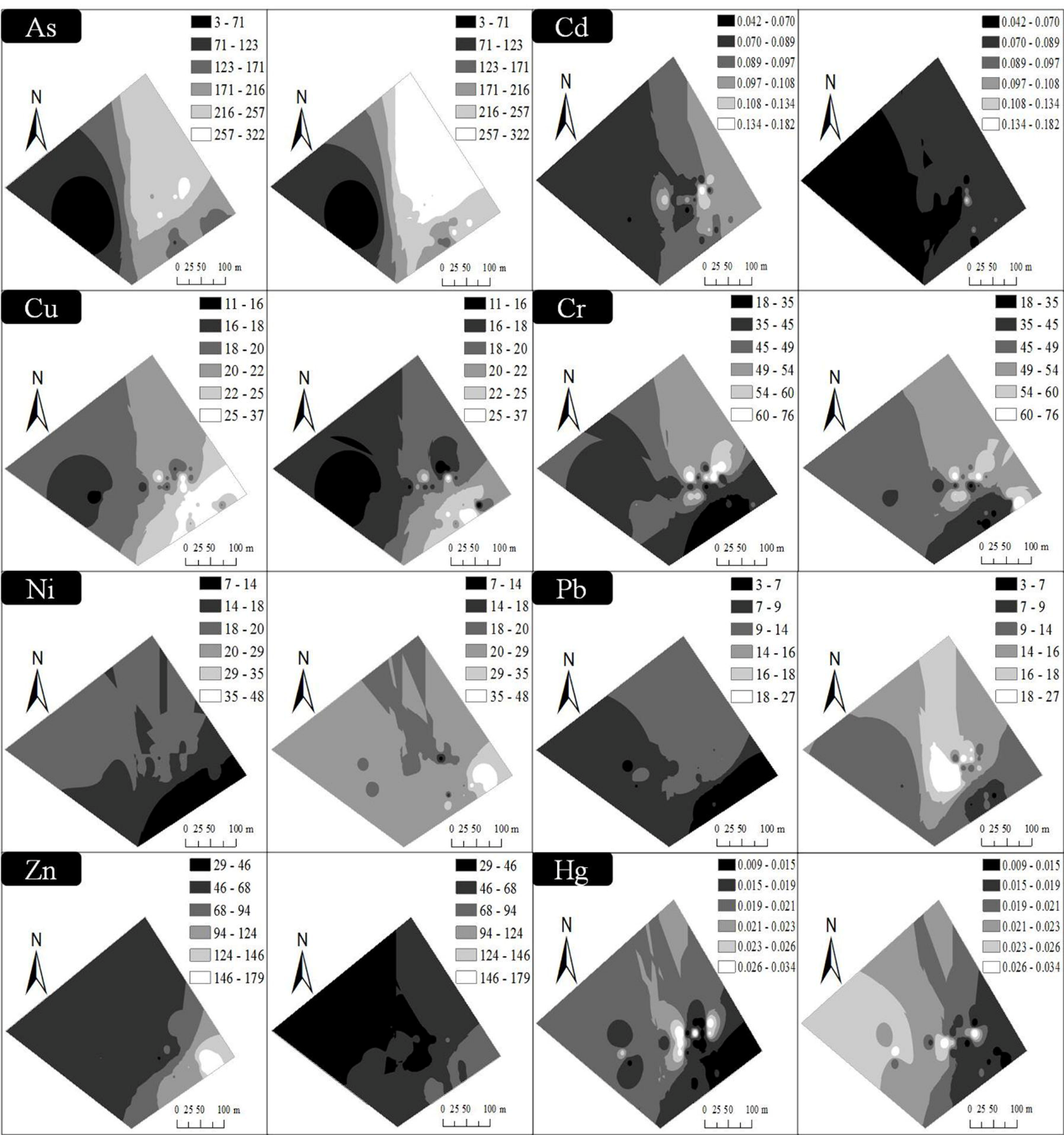


**FIGURE S2** The spatial distribution of heavy metals at different depths in soil. (Left presents 10-cm-depth soil; right presents 50-cm-depth soil)


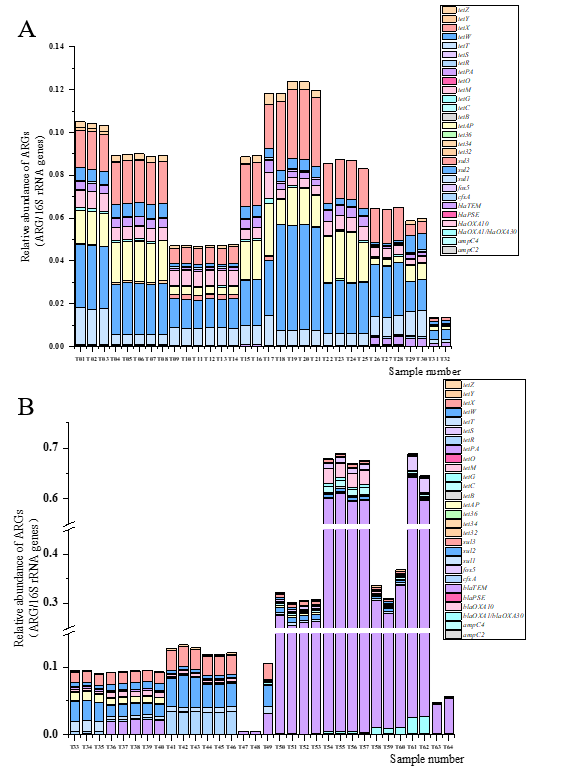


**FIGURE S3** The Relative abundance of ARGs in the samples of soil (A) 10-cm-depth soil samples and (B) 50-cm-depth soil samples


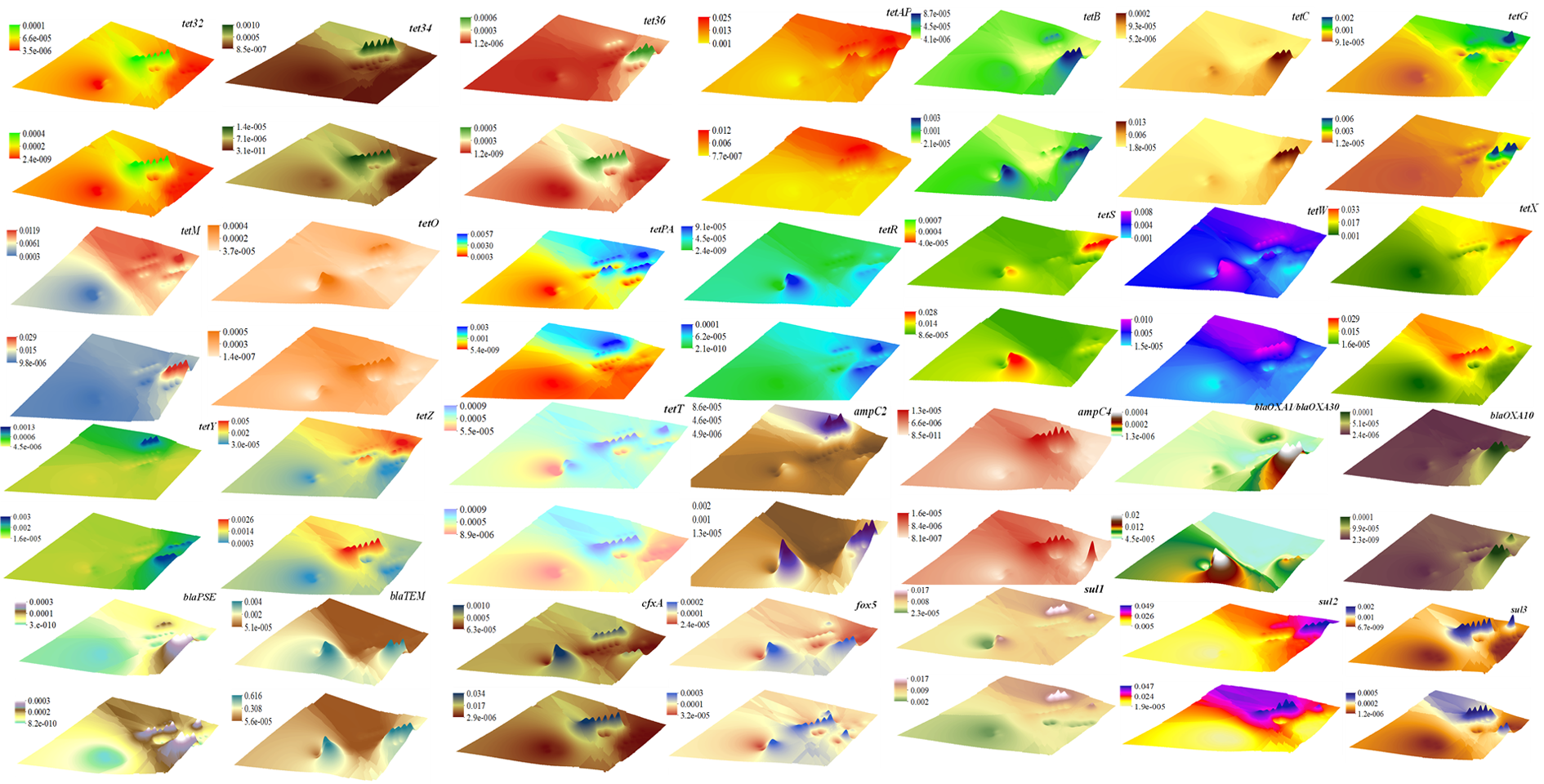


**FIGURE S4** The Spatial distribution of the total 28 tetracyclines, β-lactams and sulfonamides ARGs at different depths of the soil samples (top maps represented the 10-cm-depth soil, and bottom maps represent the 50-cm-depth soil)


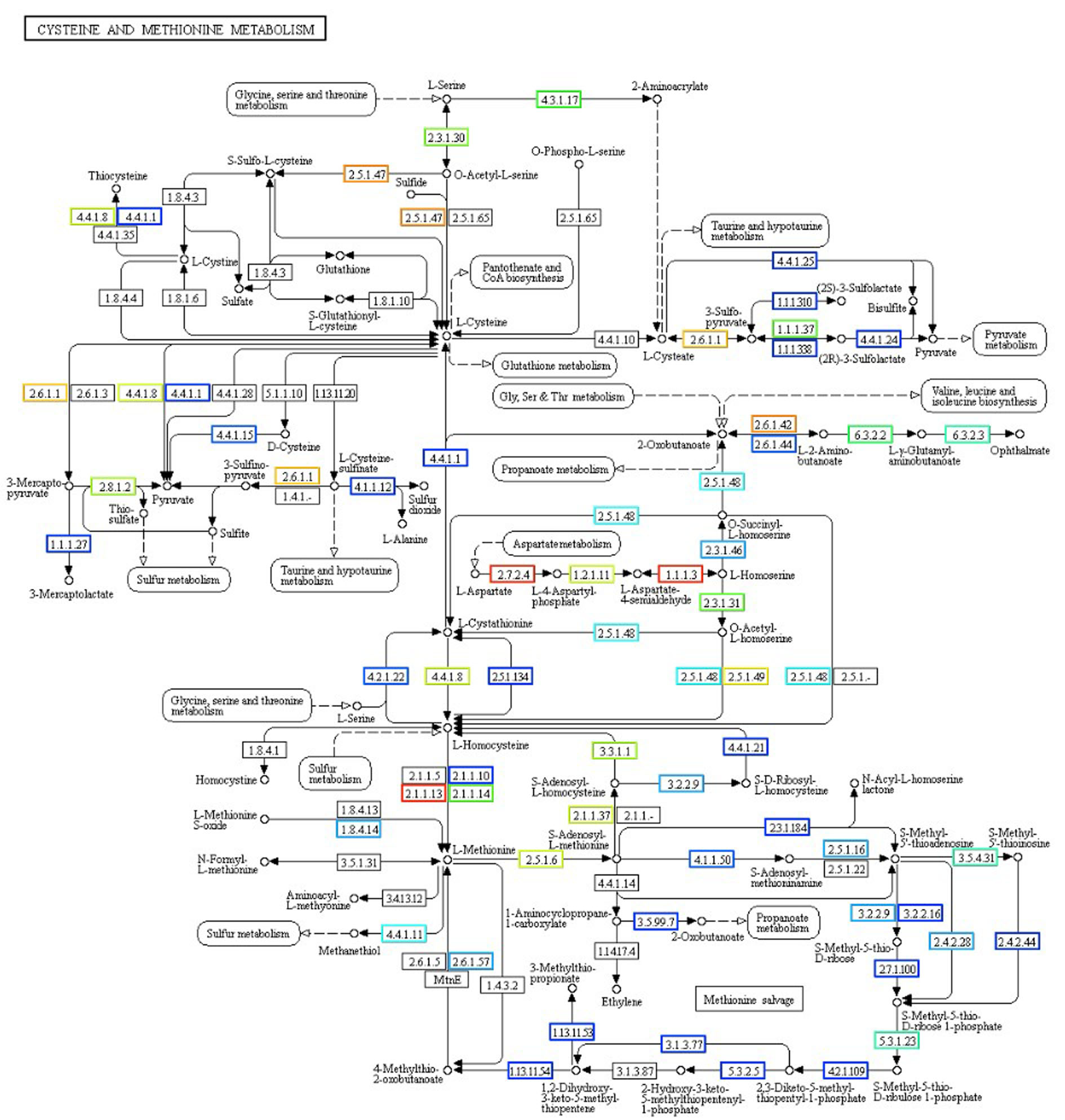


**FIGURE S5** The bioenzymes expression of amino acid metabolism path way in metabolism

TABLE S3 Total PCA variance of 28 tetracyclines, β-lactams and sulfonamides ARGs relative abundance in soil samples

| Principal component number | Initial eigenvalue λ | | | Extraction sums of squared loadings | | |
| --- | --- | --- | --- | --- | --- | --- |
|  | Total | Percent of variance (%) | Cumulative (%) | Total | Percent of variance (%) | Cumulative (%) |
| 1 | 38.5 | 60.1 | 60.1 | 38.5 | 60.1 | 60.1 |
| 2 | 19.3 | 30.1 | 90.2 | 19.3 | 30.1 | 90.2 |
| 3 | 2.94 | 4.59 | 94.8 | 2.94 | 4.59 | 94.8 |
| 4 | 1.64 | 2.57 | 97.3 | 1.64 | 2.57 | 97.3 |
| 5 | 0.663 | 1.04 | 98.4 |  |  |  |
| 6 | 0.528 | 0.826 | 99.2 |  |  |  |
| 7 | 0.312 | 0.488 | 99.7 |  |  |  |
| 8 | 0.132 | 0.206 | 99.9 |  |  |  |
| 9 | 0.042 | 0.066 | 100 |  |  |  |
| 10 | 0.006 | 0.010 | 100 |  |  |  |
| 11 | 0.005 | 0.008 | 100 |  |  |  |
| 12 | 0.003 | 0.005 | 100 |  |  |  |
| 13 | 0.002 | 0.004 | 100 |  |  |  |
| 14 | 0.002 | 0.003 | 100 |  |  |  |
| 15 | 0.002 | 0.003 | 100 |  |  |  |
| 16 | 0.001 | 0.002 | 100 |  |  |  |
| 17 | 0.001 | 0.001 | 100 |  |  |  |
| 18 | 0.0004 | 5.78E-04 | 100 |  |  |  |
| 19 | 0.0002 | 2.76E-04 | 100 |  |  |  |
| 20 | 8.45E-05 | 1.32E-04 | 100 |  |  |  |
| 21 | 4.33E-05 | 6.77E-05 | 100 |  |  |  |
| 22 | 1.29E-05 | 2.02E-05 | 100 |  |  |  |
| 23 | 1.16E-05 | 1.81E-05 | 100 |  |  |  |
| 24 | 2.97E-06 | 4.64E-06 | 100 |  |  |  |
| 25 | 1.96E-06 | 3.07E-06 | 100 |  |  |  |
| 26 | 7.43E-07 | 1.16E-06 | 100 |  |  |  |
| 27 | 4.49E-07 | 7.02E-07 | 100 |  |  |  |
| 28 | 2.71E-31 | 4.24E-31 | 100 |  |  |  |

TABLE S4 The correlation coefficients of soil ARGs in the dairy farms

|  | ***ampC2*** | ***ampC4*** | ***blaOXA1*** | ***blaOXA10*** | ***blaPSE*** | ***blaTEM*** | ***cfxA*** | ***fox5*** | ***sul1*** | ***sul2*** | ***sul3*** | ***tet32*** | ***tet34*** | ***tet36*** | ***tetAP*** | ***tetB*** | ***tetC*** | ***tetG*** | ***tetM*** | ***tetO*** | ***tetPA*** | ***tetR*** | ***tetS*** | ***tetT*** | ***tetW*** | ***tetX*** | ***tetY*** | ***tetZ*** |
| --- | --- | --- | --- | --- | --- | --- | --- | --- | --- | --- | --- | --- | --- | --- | --- | --- | --- | --- | --- | --- | --- | --- | --- | --- | --- | --- | --- | --- |
| ***ampC2*** | **1.000** |  |  |  |  |  |  |  |  |  |  |  |  |  |  |  |  |  |  |  |  |  |  |  |  |  |  |  |
| ***ampC4*** | **-0.135** | **1.000** |  |  |  |  |  |  |  |  |  |  |  |  |  |  |  |  |  |  |  |  |  |  |  |  |  |  |
| ***blaOXA1*** | **0.663**** | **0.044** | **1.000** |  |  |  |  |  |  |  |  |  |  |  |  |  |  |  |  |  |  |  |  |  |  |  |  |  |
| ***blaOXA10*** | **0.198** | **0.010** | **0.272*** | **1.000** |  |  |  |  |  |  |  |  |  |  |  |  |  |  |  |  |  |  |  |  |  |  |  |  |
| ***blaPSE*** | **-0.156** | **0.272*** | **0.038** | **0.776**** | **1.000** |  |  |  |  |  |  |  |  |  |  |  |  |  |  |  |  |  |  |  |  |  |  |  |
| ***blaTEM*** | **0.989**** | **-0.156** | **0.643**** | **0.210** | **-0.166** | **1.000** |  |  |  |  |  |  |  |  |  |  |  |  |  |  |  |  |  |  |  |  |  |  |
| ***cfxA*** | **-0.144** | **0.625**** | **-0.060** | **0.141** | **0.462**** | **-0.157** | **1.000** |  |  |  |  |  |  |  |  |  |  |  |  |  |  |  |  |  |  |  |  |  |
| ***fox5*** | **0.226** | **0.362**** | **0.228** | **0.411**** | **0.520**** | **0.197** | **0.528**** | **1.000** |  |  |  |  |  |  |  |  |  |  |  |  |  |  |  |  |  |  |  |  |
| ***sul1*** | **-0.384**** | **0.086** | **-0.283*** | **0.045** | **0.353**** | **-0.429**** | **0.049** | **0.251*** | **1.000** |  |  |  |  |  |  |  |  |  |  |  |  |  |  |  |  |  |  |  |
| ***sul2*** | **-0.492**** | **0.142** | **-0.377**** | **-0.033** | **0.324**** | **-0.518**** | **0.465**** | **0.033** | **0.525**** | **1.000** |  |  |  |  |  |  |  |  |  |  |  |  |  |  |  |  |  |  |
| ***sul3*** | **-0.182** | **0.531**** | **-0.107** | **-0.207** | **-0.095** | **-0.175** | **-0.071** | **-0.041** | **0.171** | **-0.114** | **1.000** |  |  |  |  |  |  |  |  |  |  |  |  |  |  |  |  |  |
| ***tet32*** | **-0.185** | **0.777**** | **-0.080** | **0.051** | **0.392**** | **-0.196** | **0.952**** | **0.520**** | **0.069** | **0.411**** | **0.191** | **1.000** |  |  |  |  |  |  |  |  |  |  |  |  |  |  |  |  |
| ***tet34*** | **-0.149** | **-0.096** | **-0.101** | **-0.206** | **-0.210** | **-0.155** | **-0.086** | **-0.208** | **-0.140** | **0.085** | **-0.141** | **-0.083** | **1.000** |  |  |  |  |  |  |  |  |  |  |  |  |  |  |  |
| ***tet36*** | **-0.280*** | **0.302*** | **-0.162** | **0.106** | **0.545**** | **-0.297*** | **0.679**** | **0.283*** | **0.218** | **0.527**** | **-0.146** | **0.614**** | **-0.110** | **1.000** |  |  |  |  |  |  |  |  |  |  |  |  |  |  |
| ***tetAP*** | **-0.395**** | **-0.369**** | **-0.289*** | **-0.323**** | **-0.182** | **-0.418**** | **-0.304*** | **-0.530**** | **0.376**** | **0.439**** | **-0.093** | **-0.333**** | **0.432**** | **0.197** | **1.000** |  |  |  |  |  |  |  |  |  |  |  |  |  |
| ***tetB*** | **0.957**** | **-0.154** | **0.564**** | **0.128** | **-0.232** | **0.966**** | **-0.152** | **0.152** | **-0.443**** | **-0.511**** | **-0.178** | **-0.186** | **-0.174** | **-0.316*** | **-0.412**** | **1.000** |  |  |  |  |  |  |  |  |  |  |  |  |
| ***tetC*** | **0.890**** | **-0.204** | **0.308*** | **0.112** | **-0.197** | **0.910**** | **-0.143** | **0.146** | **-0.388**** | **-0.427**** | **-0.151** | **-0.183** | **-0.134** | **-0.259*** | **-0.365**** | **0.897**** | **1.000** |  |  |  |  |  |  |  |  |  |  |  |
| ***tetG*** | **0.563**** | **-0.183** | **-0.048** | **0.002** | **-0.078** | **0.563**** | **-0.042** | **0.125** | **0.018** | **-0.052** | **-0.049** | **-0.082** | **-0.062** | **-0.090** | **-0.079** | **0.556**** | **0.776**** | **1.000** |  |  |  |  |  |  |  |  |  |  |
| ***tetM*** | **0.542**** | **-0.115** | **-0.064** | **-0.162** | **-0.212** | **0.552**** | **-0.126** | **-0.026** | **-0.119** | **-0.137** | **0.115** | **-0.098** | **-0.003** | **-0.096** | **-0.008** | **0.566**** | **0.800**** | **0.845**** | **1.000** |  |  |  |  |  |  |  |  |  |
| ***tetO*** | **-0.070** | **0.521**** | **0.141** | **0.073** | **0.382**** | **-0.120** | **0.778**** | **0.548**** | **0.359**** | **0.424**** | **-0.158** | **0.732**** | **-0.084** | **0.584**** | **-0.068** | **-0.094** | **-0.205** | **-0.119** | **-0.172** | **1.000** |  |  |  |  |  |  |  |  |
| ***tetPA*** | **-0.416**** | **-0.315*** | **-0.301*** | **-0.312*** | **-0.155** | **-0.441**** | **-0.258*** | **-0.508**** | **0.400**** | **0.428**** | **-0.062** | **-0.281*** | **0.437**** | **0.220** | **0.987**** | **-0.433**** | **-0.383**** | **-0.074** | **-0.005** | **-0.020** | **1.000** |  |  |  |  |  |  |  |
| ***tetR*** | **0.161** | **-0.130** | **-0.020** | **0.670**** | **0.524**** | **0.162** | **-0.036** | **0.386**** | **0.367**** | **0.063** | **-0.180** | **-0.106** | **-0.247*** | **0.127** | **-0.059** | **0.120** | **0.209** | **0.285*** | **0.113** | **0.112** | **-0.058** | **1.000** |  |  |  |  |  |  |
| ***tetS*** | **0.864**** | **-0.095** | **0.845**** | **0.029** | **-0.238** | **0.848**** | **-0.106** | **0.229** | **-0.342**** | **-0.410**** | **-0.164** | **-0.123** | **-0.121** | **-0.248*** | **-0.322**** | **0.827**** | **0.616**** | **0.286*** | **0.253*** | **0.051** | **-0.347**** | **-0.056** | **1.000** |  |  |  |  |  |
| ***tetT*** | **-0.385**** | **0.655**** | **-0.163** | **0.005** | **0.472**** | **-0.417**** | **0.621**** | **0.462**** | **0.579**** | **0.538**** | **0.449**** | **0.716**** | **-0.181** | **0.670**** | **0.063** | **-0.445**** | **-0.391**** | **-0.107** | **-0.095** | **0.638**** | **0.106** | **0.133** | **-0.301*** | **1.000** |  |  |  |  |
| ***tetW*** | **-0.271*** | **0.071** | **-0.190** | **-0.224** | **-0.115** | **-0.312*** | **0.131** | **-0.212** | **0.301*** | **0.401**** | **-0.154** | **0.096** | **0.268*** | **0.087** | **0.490**** | **-0.203** | **-0.286*** | **-0.140** | **-0.039** | **0.513**** | **0.511**** | **0.020** | **-0.235** | **0.154** | **1.000** |  |  |  |
| ***tetX*** | **-0.458**** | **0.147** | **-0.344**** | **-0.011** | **0.305*** | **-0.479**** | **0.502**** | **-0.059** | **0.383**** | **0.957**** | **-0.144** | **0.443**** | **0.175** | **0.542**** | **0.475**** | **-0.456**** | **-0.393**** | **-0.041** | **-0.092** | **0.428**** | **0.479**** | **0.020** | **-0.380**** | **0.466**** | **0.469** | **1.000** | **-** |  |
| ***tetY*** | **0.558**** | **-0.052** | **0.290*** | **0.561**** | **0.229** | **0.576**** | **-0.053** | **0.198** | **-0.074** | **-0.311*** | **-0.196** | **-0.123** | **-0.135** | **-0.127** | **-0.291*** | **0.540**** | **0.477**** | **0.202** | **0.105** | **0.011** | **-0.295*** | **0.379**** | **0.289*** | **-0.269*** | **-0.120** | **-0.307*** | **1.000** | **-** |
| ***tetZ*** | **-0.261*** | **0.060** | **-0.209** | **-0.289*** | **-0.153** | **-0.274*** | **0.272*** | **-0.122** | **0.397**** | **0.668**** | **0.041** | **0.253*** | **0.330**** | **0.094** | **0.473**** | **-0.294*** | **-0.220** | **0.095** | **0.028** | **0.297*** | **0.483**** | **-0.195** | **-0.183** | **0.291*** | **0.428** | **0.682**** | **-0.174** | **1.000** |

Two asterisks indicated significant correlation P<0.01, bilateral); one asterisk indicates significant correlation (P<0.05, bilateral).
